# Emerging perspectives on once-weekly insulins in type 1 and type 2 diabetes: a mini-review

**DOI:** 10.3389/fendo.2025.1656884

**Published:** 2025-08-27

**Authors:** Damien Denimal

**Affiliations:** Université Bourgogne Europe, CHU Dijon Bourgogne, Department of Clinical Biochemistry, INSERM, CTM UMR 1231, PADYS team, Dijon, France

**Keywords:** diabetes mellitus, insulin icodec, insulin efsitora, long-acting insulin, glycemic control

## Abstract

The development of once-weekly basal insulin analogues, such as insulin icodec and efsitora alfa, represents a promising strategy to reduce injection burden and improve adherence in diabetes management. This mini-review summarizes the recent findings from clinical trials evaluating once-weekly insulin therapies in both type 1 and type 2 diabetes. In type 1 diabetes, available data remain limited; however, the ONWARDS 6 and QWINT-5 trials demonstrated that once-weekly icodec and efsitora, respectively, achieved comparable reductions in HbA1c to once-daily insulin degludec, when used in combination with prandial insulin. In type 2 diabetes, accumulating evidences from randomized clinical trials supports the efficacy of once-weekly icodec and efsitora, showing non-inferiority—and in some cases, superiority—compared to once-daily basal insulin, both in insulin-naïve individuals and in those previously treated with insulin. Safety profiles of once-weekly insulins in type 2 diabetes are reassuring, with similar rates of clinically significant and severe hypoglycemia compared to once-daily regimens. In contrast, trials in type 1 diabetes reported higher hypoglycemia rates with once-weekly insulins. Recent findings from the COMBINE program demonstrated that the fixed-ratio combination of icodec and semaglutide (IcoSema) produced superior HbA1c reductions compared to either agent alone, though not superior to a basal-bolus regimen with glargine and aspart insulin. However, several important questions remain to be addressed regarding once-weekly insulins, including their long-term efficacy on cardiovascular outcomes and overall long-term safety.

## 1 Introduction

Type 1 diabetes necessitates lifelong basal and prandial insulin therapy to maintain glycemic control, typically requiring multiple daily injections—a factor that significantly impacts quality of life. Type 2 diabetes is characterized by insulin resistance and the gradual decline of pancreatic β-cell function. For many patients, this trajectory leads to the need for insulin therapy—approximately one in three individuals with type 2 diabetes will require insulin therapy within seven years of diagnosis (1). While the therapeutic landscape has expanded with several non-insulin glucose-lowering agents, real-world data show that insulin use remains the cornerstone of treatment for many individuals with type 2 diabetes. Unfortunately, psychological and practical barriers—most notably the fear of hypoglycemia and the burden of daily injections—often discourage timely insulin initiation (2).

To address these challenges, the development of once-weekly basal insulin analogues has emerged as a promising strategy to reduce treatment burden and improve adherence. These novel formulations use chemical modifications to reduce insulin receptor affinity, resulting in slower clearance and enabling a weekly dosing schedule.

Recent advances have brought two contenders to the forefront: insulin efsitora alfa (Eli Lilly™) and insulin icodec (Novo Nordisk™), the latter also studied in combination with semaglutide. This mini-review synthetizes the latest findings from six phase 3 trials published in 2025—QWINT-1, QWINT-3 and QWINT-4 for efsitora and COMBINE 1, COMBINE 2 and COMBINE 3 for icodec-semaglutide—to offer an updated perspective on the clinical promise of once-weekly insulins (3–7). **Table 1** summarizes the key characteristics and outcomes of clinical trials involving once-weekly insulins in both type 1 and type 2 diabetes.

**Table 1:** Summary of randomized controlled trials investigating once-weekly insulin in type 1 and type 2 diabetes.

Trial, year of publication (ClinicalTrials.gov ID)	Baseline treatment	Intervention (number of randomized participants)	Control (number of randomized participants)	Trial duration	Main findings
Type 1 diabetes					
ONWARDS 6, 2023 (15) (NCT04848480)	Daily basal + prandial insulin	Icodec + insulin aspart (n=290)	Degludec + insulin aspart (n=292)	52 weeks	Similar HbA1c reduction between groups at week 26 (ETD: 0.05% [95% CI -0.13 to 0.23]). Lower HbA1c reduction at week 52 (ETD: 0.17% [95% CI 0.02 to 0.31]). Higher rate of level 2 or level 3 hypoglycemia with icodec (ERR 1.9 [95% CI 1.5 to 2.3]).
Kazda et al., 2023 (19) (NCT04450407)	Daily basal + prandial insulin	Efsitora + previous prandial insulin (n=139)	Degludec + previous prandial insulin (n=126)	26 weeks	Higher HbA1_C_ reduction with efsitora at week 26 (ETD: 0.17% [95% CI 0.01 to 0.32]). Similar rate of level 1 (ERR: 1.06 [95% CI 0.89 to 1.27]) and level 2 hypoglycemia (ERR: 1.09 [95% CI 0.86 to 1.39]).
QWINT-5, 2024 (20) (NCT05463744)	Daily basal + prandial insulin	Efsitora + insulin lispro (n=343)	Degludec + insulin lispro (n=349)	52 weeks	Similar HbA1c reduction at week 26 (ETD: -0.052% [95% CI -0.077 to 0.181]) and at week 52 (ETD: 0.024% [95% CI -0.110 to 0.157]). Higher rate of level 2 or level 3 hypoglycemia with efsitora (ERR 1.21 [95% CI 1.04 to 1.41]).
Type 2 diabetes					
Icodec					
Rosenstock et al., 2020 (21) (NCT03751657)	Insulin-naive	Icodec (n=125)	Glargine (n=122)	26 weeks	Similar HbA1c reduction at week 26 (ETD: -0.18% [95% CI -0.38 to 0.02]). Similar rate of level 2 or level 3 hypoglycemia (ERR 1.09 [95% CI 0.45 to 2.65]).
Bajaj et al., 2021 (22) (NCT03922750)	Daily basal insulin	Icodec (n=54)	Glargine (n=50)	16 weeks	Similar HbA1c reduction at week 16 (ETD: -0.23% [95% CI -0.49 to 0.02]). Similar rate of level 2 or level 3 hypoglycemia (ERR 0.94 [95% CI 0.44 to 1.98]).
Lingvay et al., 2021 (23) (NCT03951805)	Insulin-naive	Icodec (n=51)	Glargine (n=51)	16 weeks	Similar HbA1c reduction at week 16 (ETD: -0.20% [95% CI -0.42 to 0.02]). 0.15 level 2 or level 3 hypoglycemic events per patient-year vs. 0 in the glargine group.
ONWARDS 1, 2023 (12) (NCT04460885)	Insulin-naive	Icodec (n=492)	Glargine U100 (n=492)	78 weeks	Higher HbA1_C_ reduction with icodec at week 52 (ETD: -0.19% [95% CI -0.36 to -0.03]) and at week 78 (ETD: -0.11% [95% CI -0.22 to -0.00]). Similar rate of level 2 or level 3 hypoglycemia between groups at week 52 (ERR 1.64 [95% CI 0.98 to 2.75]) and higher rate at week 89 (ERR 1.63 [95% CI 1.02 to 2.61]).
ONWARDS 2, 2023 (13) (NCT04770532)	Daily basal insulin	Icodec (n=263)	Degludec (n=263)	26 weeks	Higher HbA1_C_ reduction with icodec at week 26 (ETD: -0.22% [95% CI -0.37 to -0.08]). Similar rate of level 2 or level 3 hypoglycemia (ERR 1.93 [95% CI 0.93 to 4.02]).
ONWARDS 3, 2023 (10) (NCT04795531)	Insulin-naive	Icodec (n=294)	Degludec (n=294)	26 weeks	Higher HbA1_C_ reduction with icodec at week 26 (ETD: -0.2% [95% CI -0.3 to -0.1]). Higher rate of level 2 or level 3 hypoglycemia at week 26 (ERR 3.12 [95% CI 1.30 to 7.51]) and similar rate at week 31 (ERR 1.82 [95% CI 0.87 to 3.80]).
ONWARDS 4, 2023 (14) (NCT04880850)	Daily basal + prandial insulin	Icodec (n=291)	Glargine U100 + prandial aspart insulin (n=291)	26 weeks	Similar HbA1c reduction at week 26 (ETD: -0.02% [95% CI -0.11 to 0.15]). Similar rate of level 2 or level 3 hypoglycemia (ERR 0.99 [95% CI 0.73 to 1.33]).
ONWARDS 5, 2023 (11) (NCT04760626)	Insulin-naive	Icodec (n=542)	Once-daily analogue (degludec, glargine) (n=543)	52 weeks	Higher HbA1_C_ reduction with icodec at week 52 (ETD: -0.38% [95% CI -0.66 to -0.09]). Similar rate of level 2 or level 3 hypoglycemia (ERR 1.17 [95% CI 0.73 to 1.86]).
Efsitora alfa					
QWINT-1, 2025 (8) (NCT05662332)	Insulin-naive	Efsitora alfa (n=397)	Glargine U100 (n=398)	52 weeks	Similar HbA1c reduction at week 52 (ETD: -0.03% [95% CI -0.18 to 0.12]). Lower rate of level 2 or level 3 hypoglycemia with efsitora (ERR 0.57 [95% CI 0.39 to 0.84]).
QWINT-2, 2024 (3) (NCT05362058)	Insulin-naive	Efsitora alfa (n=466)	Degludec (n=462)	52 weeks	Similar HbA1c reduction at week 26 (ETD: -0.09% [95% CI -0.22 to 0.04]). Similar rate of level 2 or level 3 hypoglycemia (ERR 1.30 [95% CI 0.94 to 1.78]).
QWINT-3, 2025 (4) (NCT05275400)	Daily basal insulin	Efsitora alfa (n=655)	Degludec (n=331)	78 weeks	Similar HbA1c reduction at week 26 (ETD: -0.09% [95% CI -0.19 to 0.01]) and at week 78 (ETD: -0.11% [95% CI -0.24 to 0.01]). Similar rate of level 2 or level 3 hypoglycemia (ERR 1.14 [95% CI 0.83 to 1.56]).
QWINT-4, 2025 (5) (NCT05462756)	Daily basal + prandial insulin	Efsitora alfa + prandial insulin lispro (n=365)	Glargine + prandial insulin lispro (n=365)	26 weeks	Similar HbA1c reduction at week 52 (ETD: -0.01% [95% CI -0.14 to 0.12]). Similar rate of level 2 or level 3 hypoglycemia (ERR 1.11 [95% CI 0.85 to 1.44]).
IcoSema					
COMBINE 1, 2025 (7) (NCT05352815)	Daily basal insulin	IcoSema (n=646)	Icodec (n=645)	52 weeks	Higher HbA1_C_ reduction with IcoSema at week 52 (ETD: -0.66% [95% CI -0.76 to -0.57]). Lower rate of level 2 or level 3 hypoglycemia with IcoSema (ERR 0.22 [95% CI 0.14 to 0.36]).
COMBINE 3, 2025 (6) (NCT05013229)	Daily basal insulin	IcoSema (n=340)	Glargine U100 + prandial insulin aspart (n=339)	52 weeks	Similar HbA1c reduction at week 52 (ETD: -0.06% [95% CI -0.22 to 0.09]). Lower rate of level 2 or level 3 hypoglycemia with IcoSema (ERR 0.12 [95% CI 0.08 to 0.17]).
COMBINE 2, 2025 (17) (NCT05259033)	Insulin-naive on GLP-1 RA	IcoSema (n=342)	Semaglutide (n=341)	52 weeks	Higher HbA1_C_ reduction with IcoSema at week 52 (ETD: -0.44% [95% CI -0.56 to -0.33]). Similar rate of level 2 or level 3 hypoglycemia (ERR 1.20 [95% CI 0.53 to 2.69]).

CI, confidence interval; ERR, estimated rate ratio; ETD, estimated treatment difference; GLP-1 RA, glucagon-like peptide-1 receptor agonists.

## 2 Efsitora alfa

Insulin efsitora alfa represents a new generation of basal insulin therapy, uniquely engineered as a fusion protein combining the Fc region of human IgG with a single-chain insulin variant. Its receptor binding is about 100 times weaker than that of natural insulin, enabling a pharmacokinetic profile suitable for once-weekly administration.

Over the past year, a robust body of evidence has emerged from the QWINT phase 3 clinical trial programme (QWINT-1 to QWINT-4 in type 2 diabetes and QWINT-5 in type 1 diabetes), collectively demonstrating that efsitora is an effective alternative to conventional daily insulins.

The first published trial, QWINT-2 (NCT05362058), compared once-weekly efsitora to daily degludec in 928 insulin-naive adults with type 2 diabetes (3). After 52 weeks, efsitora achieved non-inferior HbA1c reduction (-1.26% vs -1.17%; difference -0.09%, 95% confidence interval [CI] -0.22 to 0.04) and showed a slight advantage in time-in-range (64.3% vs 61.2%), with comparable hypoglycemia and adverse event profiles. Interestingly, subgroup analyses confirmed noninferiority in those using or not using GLP-1 receptor agonists.

The QWINT-1 trial (NCT05662332), published in June 2025, confirmed these findings against daily insulin glargine (8). In 795 adults with type 2 diabetes naïve to insulin therapy, efsitora again achieved non-inferior HbA1c reduction (-1.19% vs. -1.16%; between-group difference -0.03%, 95% CI -0.18 to 0.12) at 52 weeks, but with fewer clinically significant or severe hypoglycemia episodes (0.50 vs. 0.88 events/person-year; rate ratio 0.57, 95% CI 0.39 to 0.84). However, reescalation of efsitora doses after a dose reduction due to hypoglycemia was prohibited, likely reducing the incidence of hypoglycemia in this group. Patient-reported outcomes favored efsitora, and treatment satisfaction scores improved in both groups. Adverse event rates and body weight gain were comparable.

The QWINT-3 trial (NCT05275400), which had the longest treatment duration trial among the studies conducted on once-weekly insulins, evaluated efsitora in 986 individuals with type 2 diabetes inadequately controlled on basal insulin (4). Over 78 weeks, efsitora was non-inferior to degludec in HbA1c reduction (-0.81% vs. -0.72% at week 26; estimated treatment difference -0.09%, 95% CI -0.19 to 0.01). Continuous glucose monitoring indicated similar improvements in time-in-range between groups, and patient-reported outcomes favored efsitora in terms of treatment satisfaction. Rates of clinically significant or severe hypoglycaemia and serious adverse events were similar. However, slight hypoglycemia was more frequent in the efsitora group. Despite a higher incidence of mild side effects such as headache and anaemia, no treatment-related deaths occurred.

Finally, the QWINT-4 trial (NCT05462756) extended the evidence base to people with type 2 diabetes already on basal-bolus therapy. In 730 participants, efsitora was non-inferior to glargine in HbA1c reduction over 26 weeks (-1.01% vs -1.00%; treatment difference -0.01%, 95% CI -0.14 to 0.12). It also showed numerically fewer nocturnal hypoglycemic events. Continuous glucose monitoring revealed similar time-in-range, with a slight advantage for efsitora during daytime hours. Once again, patient preference strongly leaned toward the simplicity of weekly dosing.

In type 1 diabetes, available data on efsitora alfa remain limited. The QWINT-5 trial (NCT05463744) demonstrated comparable reductions in HbA1c levels at 52 weeks between efsitora and degludec, both administered alongside with insulin lispro (-0.39% vs. -0.44%; treatment difference 0.024% [95% CI -0.110 to 0.157]). Notably, treatment satisfaction scores favored efsitora over degludec. However, a major safety concern emerged regarding hypoglycemia: efsitora was associated with higher rates of level 1 (<70 mg/dL), combined level 2 (<54 mg/dL) and level 3 (severe episodes requiring external assistance) hypoglycemia, including a greater non-nocturnal level 3 events. Interestingly, the incidence of nocturnal level 1 hypoglycemia with efsitora was lower in patients with type 2 diabetes than in those with type 1 diabetes (9).

Taken together, the QWINT trials position efsitora as a clinically effective once-weekly basal insulin that addresses common barriers to insulin initiation and persistence—namely, injection burden, and treatment satisfaction. Nevertheless, safety concerns remain regarding the risk of hypoglycemia in type 1 diabetes, which warrants further investigation. Additionally, all of these trials were open-label, which may introduce biases, including those related to the self-reporting of hypoglycemic events.

## 3 Icodec

Insulin icodec, another once-weekly basal insulin analogue, represents a significant pharmacological advancement owing to its albumin-binding fatty acid modifications and reduced affinity for the insulin receptor. These structural optimizations extend its half-life to approximately 196 hours, thereby enabling sustained glycemic control with a single weekly injection.

In type 2 diabetes, the efficacy and safety of icodec have been robustly evaluated through the ONWARDS phase 3 trials, published in 2023. In insulin-naïve individuals with type 2 diabetes (ONWARDS 1, 3, and 5), once-weekly icodec demonstrated superior HbA1c reductions compared to degludec (treatment difference -0.2% at week 26, 95% CI -0.3 to -0.1), glargine (treatment difference -0.19% at week 52, 95% CI -0.36 to -0.03), and to once-daily basal insulin analogues (treatment difference -0.38% at week 52, 95% CI -0.66 to -0.09) (10–12). In patients previously treated with insulin (ONWARDS 2 and 4), icodec was superior to degludec (treatment difference -0.22%, 95% CI -0.37 to -0.08) and non-inferior to glargine (treatment difference -0.02%, 95% CI -0.11 to 0.15) in HbA1c reduction after 26 weeks of treatment (13, 14).

In type 1 diabetes, available evidence is currently limited to the ONWARDS 6 trial (NCT04848480) (15). This study demonstrated comparable reductions in HbA1c levels at 26 weeks between icodec and degludec, both administered alongside with insulin aspart (-0.47% vs. -0.51%; treatment difference 0.05% [95% CI -0.13 to 0.23]) (15). However, at 52 weeks, icodec was less effective than degludec in lowering HbA1c (-0.37% vs. -0.54%; treatment difference 0.17% [95% CI 0.02 to 0.31]). Consistent with findings for efsitora in patients with type 1 diabetes, a notable safety concern with icodec was a higher incidence of hypoglycemia compared to degludec. Specifically, the percentage of time spent with blood glucose levels below 54 mg/dL was significantly greater with icodec (1.0% vs. 0.7%). A *post-hoc* analysis of the ONWARDS 6 trial reported no significant increase in the odds of hypoglycemia attributed to physical activity with once-weekly icodec compared to degludec (16).

The once-weekly fixed-ratio combination IcoSema (icodec + semaglutide) has recently emerged as a compelling intensification strategy. The first findings of phase 3 trials using IcoSema have just been published in 2025. In the COMBINE 1 trial (NCT05352815), IcoSema showed superior efficacy on HbA1c reduction compared to icodec alone (-1.55% vs. -0.89% at 52 weeks; treatment difference -0.66%, 95% CI -0.76 to -0.57) in 1291 adults with type 2 diabetes inadequately controlled on daily basal insulin (7). IcoSema also led to significant weight loss (-3.7 kg vs. +1.9 kg; treatment difference -5.59 kg) and a lower rate of clinically significant or severe hypoglycaemia (0.14 vs. 0.63 events/person-year; rate ratio 0.22). Time-in-range was notably greater (75.9% vs. 61.9%), and more participants achieved HbA1c <7.0% without weight gain or hypoglycaemia. While mild gastrointestinal adverse events were more frequent, overall safety was comparable, with no new safety concerns.

In the COMBINE 2 trial (NCT05259033), IcoSema also outperformed once-weekly semaglutide in 683 patients with type 2 diabetes inadequately controlled on GLP-1 receptor agonist, achieving a greater HbA1c reduction at 52 weeks (-1.35% vs. -0.90%; treatment difference -0.44%, 95% CI -0.56 to -0.33) (17). The rate of clinically significant or severe hypoglycaemia was similar between the two groups.

Finally, the COMBINE 3 trial (NCT05013229) compared once-weekly IcoSema with conventional basal-bolus therapy (glargine + insulin aspart) in 679 patients with type 2 diabetes insufficiently controlled on basal insulin (6). IcoSema achieved non-inferior HbA1c reduction (-1.47% vs. -1.40% at week 52; treatment difference -0.06%, 95% CI -0.22 to 0.09) and demonstrated superiority in multiple secondary outcomes, including substantial weight reduction (-3.56 vs. +3.16 kg; treatment difference -6.72 kg; 95% CI -7.58 to -5.86), and a significant reduction in clinically significant or severe hypoglycaemia episodes (0.21 vs. 2.23 episodes per person-year of exposure; rate ratio 0.12, 95% CI 0.08 to 0.17). Continuous glucose monitoring showed similar time-in-range, with significantly less time spent below 54 mg/dL in the IcoSema group. Again, gastrointestinal side effects were more frequent but mostly mild, and consistent with known profiles of GLP-1 receptor agonists. Patient-reported outcomes consistently favored IcoSema for convenience and satisfaction.

Together, these findings position IcoSema as a powerful, once-weekly intensification option offering superior glycaemic control, meaningful weight benefits, and a lower risk of hypoglycaemia—an appealing alternative to traditional basal-bolus therapy in the management of type 2 diabetes. Similar to the QWINT trials, the COMBINE trials were open-label, which may introduce participant bias when reporting.

## 4 Discussion

The emergence of once-weekly basal insulin analogues represents a transformative shift in the treatment landscape of type 1 and type 2 diabetes, addressing long-standing barriers to insulin treatment. Both insulin efsitora alfa and insulin icodec offer simplified regimens with promising efficacy and safety profiles, as demonstrated in multiple recent phase 3 trials conducted in patients with type 2 diabetes. However, a notable safety concern with both efsitora and icodec was a higher incidence of hypoglycemia.

Efsitora alfa has shown consistent glycaemic control across a broad range of patient populations with type 2 diabetes—from insulin-naïve individuals (QWINT-1, QWINT-2) to those previously treated with basal or basal-bolus insulin (QWINT-3, QWINT-4). Across these trials, efsitora achieved non-inferior HbA1c reductions compared to daily basal insulin analogues, with additional benefits in hypoglycaemia risk, time-in-range, and treatment satisfaction. Importantly, patient-reported outcomes favored once-weekly dosing, highlighting the potential to improve long-term adherence.

Similarly, insulin icodec has demonstrated robust efficacy, with superior glycaemic outcomes in both insulin-naïve and insulin-experienced populations with type 2 diabetes, as shown in the ONWARDS programme. The addition of semaglutide in the fixed-ratio formulation IcoSema further enhances clinical outcomes. COMBINE trials have revealed superior HbA1c reduction, greater weight loss, and fewer hypoglycaemic events compared to either icodec alone, semaglutide monotherapy, or even conventional basal-bolus regimens.

Together, these findings suggest that once-weekly insulins could redefine insulin-based management strategies in diabetes, combining clinical efficacy with a reduced treatment burden. Nevertheless, several limitations merit further consideration. The fixed weekly administration schedule limits dosing flexibility, and the initiation of such therapies requires re-education of both patients and healthcare providers.

Once-weekly insulins beyond icodec and efsitora alfa are currently in development. Notably, GZR4 has recently demonstrated a favorable safety profile in a phase 1a trial (18). Several ongoing clinical trials are expected to more precisely define the efficacy and safety of these agents. In particular, the ONWARDS 11 trial (NCT07076199) will evaluate icodec versus glargine in individuals with type 1 diabetes, while the single-arm trial NCT06807190 will assess icodec in Japanese participants irrespective of diabetes type. Additionally, the COMBINE 4 trial (NCT06269107) will compare icosema with glargine in insulin-naïve individuals with type 2 diabetes. Beyond these ongoing investigations, further research is needed to assess long-term cardiovascular outcomes and safety profiles, cost-effectiveness, real-world adherence and head-to-head comparisons between efsitora and icodec. As these therapies advance toward broader clinical use, they hold the potential to significantly improve the quality of diabetes care.

## References

[1] Gentile S Strollo F Viazzi F Russo G Piscitelli P Ceriello A. Five-year predictors of insulin initiation in people with type 2 diabetes under real-life conditions. J Diabetes Res. (2018) 2018:7153087. doi: 10.1155/2018/7153087, PMID: 30327785 PMC6169213

[2] Galdón Sanz-Pastor A Justel Enríquez A Sánchez Bao A Ampudia-Blasco FJ. Current barriers to initiating insulin therapy in individuals with type 2 diabetes. Front Endocrinol. (2024) 15:1366368. doi: 10.3389/fendo.2024.1366368, PMID: 38559691 PMC10979640

[3] Wysham C Bajaj HS Del Prato S Franco DR Kiyosue A Dahl D. Insulin Efsitora versus Degludec in Type 2 Diabetes without Previous Insulin Treatment. N Engl J Med. (2024) 391:2201–11. doi: 10.1056/NEJMoa2403953, PMID: 39254740

[4] Philis-Tsimikas A Bergenstal RM Bailey TS Jinnouchi H Thrasher JR Ilag L. Once-weekly insulin efsitora alfa versus once-daily insulin degludec in adults with type 2 diabetes currently treated with basal insulin (QWINT-3): a phase 3, randomised, non-inferiority trial. Lancet. (2025) 405:2279–89. doi: 10.1016/S0140-6736(25)01044-X, PMID: 40562047

[5] Blevins T Dahl D Pérez Manghi FC Murthy S Ortiz Carrasquillo R Li X. Once-weekly insulin efsitora alfa versus once-daily insulin glargine U100 in adults with type 2 diabetes treated with basal and prandial insulin (QWINT-4): a phase 3, randomised, non-inferiority trial. Lancet. (2025) 405:2290–301. doi: 10.1016/S0140-6736(25)01069-4, PMID: 40562048

[6] Billings LK Andreozzi F Frederiksen M Gourdy P Gowda A Ji L. Once-weekly IcoSema versus multiple daily insulin injections in type 2 diabetes management (COMBINE 3): an open-label, multicentre, treat-to-target, non-inferiority, randomised, phase 3a trial. Lancet Diabetes Endocrinol. (2025) 13:556–67. doi: 10.1016/S2213-8587(25)00052-X, PMID: 40482670

[7] Mathieu C Giorgino F Kim SG Larsen JH Philis-Tsimikas A Ramachandran A. Once−weekly IcoSema versus once−weekly insulin icodec in type 2 diabetes management (COMBINE 1): an open−label, multicentre, treat−to−target, randomised, phase 3a trial. Lancet Diabetes Endocrinol. (2025) 13:568–79. doi: 10.1016/S2213-8587(25)00096-8, PMID: 40482671

[8] Rosenstock J Bailey T Connery L Miller E Desouza C Wang Q. Weekly fixed-dose insulin efsitora in type 2 diabetes without previous insulin therapy. N Engl J Med. (2025) 393:325–35. doi: 10.1056/NEJMoa2502796, PMID: 40548694

[9] de Oliveira HM Gallo Ruelas M Flávio-Reis VHP Queiroz I Cavalcante DVS Barbosa LM. Degludec insulin versus efsitora insulin in diabetes mellitus management: A systematic review and meta-analysis. J Diabetes Complications. (2025) 39:109115. doi: 10.1016/j.jdiacomp.2025.109115, PMID: 40627908

[10] Lingvay I Asong M Desouza C Gourdy P Kar S Vianna A. Once-weekly insulin icodec vs once-daily insulin degludec in adults with insulin-naive type 2 diabetes: the ONWARDS 3 randomized clinical trial. JAMA. (2023) 330:228–37. doi: 10.1001/jama.2023.11313, PMID: 37354562 PMC10354685

[11] Bajaj HS Aberle J Davies M Donatsky AM Frederiksen M Yavuz DG. Once-weekly insulin icodec with dosing guide app versus once-daily basal insulin analogues in insulin-naive type 2 diabetes (ONWARDS 5): A randomized trial. Ann Intern Med. (2023) 176:1476–85. doi: 10.7326/M23-1288, PMID: 37748181

[12] Rosenstock J Bain SC Gowda A Jódar E Liang B Lingvay I. Weekly Icodec versus Daily Glargine U100 in Type 2 Diabetes without Previous Insulin. N Engl J Med. (2023) 389:297–308. doi: 10.1056/NEJMoa2303208, PMID: 37356066

[13] Philis-Tsimikas A Asong M Franek E Jia T Rosenstock J Stachlewska K. Switching to once-weekly insulin icodec versus once-daily insulin degludec in individuals with basal insulin-treated type 2 diabetes (ONWARDS 2): a phase 3a, randomised, open label, multicentre, treat-to-target trial. Lancet Diabetes Endocrinol. (2023) 11:414–25. doi: 10.1016/S2213-8587(23)00093-1, PMID: 37148899

[14] Mathieu C Ásbjörnsdóttir B Bajaj HS Lane W Matos ALSA Murthy S. Switching to once-weekly insulin icodec versus once-daily insulin glargine U100 in individuals with basal-bolus insulin-treated type 2 diabetes (ONWARDS 4): a phase 3a, randomised, open-label, multicentre, treat-to-target, non-inferiority trial. Lancet Lond Engl. (2023) 401:1929–40. doi: 10.1016/S0140-6736(23)00520-2, PMID: 37156252

[15] Russell-Jones D Babazono T Cailleteau R Engberg S Irace C Kjaersgaard MIS. Once-weekly insulin icodec versus once-daily insulin degludec as part of a basal-bolus regimen in individuals with type 1 diabetes (ONWARDS 6): a phase 3a, randomised, open-label, treat-to-target trial. Lancet Lond Engl. (2023) 402:1636–47. doi: 10.1016/S0140-6736(23)02179-7, PMID: 37863084

[16] Sourij H Bracken RM Carstensen L Pagliaro Rocha TM Kehlet Watt S Philis-Tsimikas A. No evidence of increased hypoglycaemia attributed to physical activity with once-weekly insulin icodec versus once-daily basal insulin degludec in type 1 diabetes: A *post hoc* analysis of ONWARDS 6. Diabetes Obes Metab. (2025) 27:2882–6. doi: 10.1111/dom.16265, PMID: 39972508 PMC11965012

[17] Lingvay I Benamar M Chen L Fu A Jódar E Nishida T. Once-weekly IcoSema versus once-weekly semaglutide in adults with type 2 diabetes: the COMBINE 2 randomised clinical trial. Diabetologia. (2025) 68:739–51. doi: 10.1007/s00125-024-06348-5, PMID: 39820580 PMC11950020

[18] Tang C Su R Wan L Zhu M Pu J Hao C. Safety, tolerability, pharmacokinetics and pharmacodynamics of GZR4, a novel once-weekly basal insulin, in healthy participants: A randomized trial. Diabetes Obes Metab. (2025) 27:2515–22. doi: 10.1111/dom.16250, PMID: 39935080

[19] Kazda CM Bue-Valleskey JM Chien J Zhang Q Chigutsa E Landschulz W. Novel once-weekly basal insulin fc achieved similar glycemic control with a safety profile comparable to insulin degludec in patients with type 1 diabetes. Diabetes Care. (2023) 46:1052–9. doi: 10.2337/dc22-2395, PMID: 36920867 PMC10154655

[20] Bergenstal RM Weinstock RS Mathieu C Onishi Y Vijayanagaram V Katz ML. Once-weekly insulin efsitora alfa versus once-daily insulin degludec in adults with type 1 diabetes (QWINT-5): a phase 3 randomised non-inferiority trial. Lancet Lond Engl. (2024) 404:1132–42. doi: 10.1016/S0140-6736(24)01804-X, PMID: 39270686

[21] Rosenstock J Bajaj HS Janež A Silver R Begtrup K Hansen MV. NN1436–4383 investigators. Once-weekly insulin for type 2 diabetes without previous insulin treatment. N Engl J Med. (2020) 383:2107–16. doi: 10.1056/NEJMoa2022474, PMID: 32960514

[22] Bajaj HS Bergenstal RM Christoffersen A Davies MJ Gowda A Isendahl J. Switching to once-weekly insulin icodec versus once-daily insulin glargine U100 in type 2 diabetes inadequately controlled on daily basal insulin: a phase 2 randomized controlled trial. Diabetes Care. (2021) 44:1586–94. doi: 10.2337/dc20-2877, PMID: 33875485 PMC8323191

[23] Lingvay I Buse JB Franek E Hansen MV Koefoed MM Mathieu C. A randomized, open-label comparison of once-weekly insulin icodec titration strategies versus once-daily insulin glargine U100. Diabetes Care. (2021) 44:1595–603. doi: 10.2337/dc20-2878, PMID: 33875484 PMC8323172

